# Revitalizing cadmium-stressed sunflower: co-composted biochar improves growth, antioxidant responses, and soil remediation efficiency

**DOI:** 10.1186/s12870-025-06906-y

**Published:** 2025-07-04

**Authors:** Muhammad Rauf, Muhammad Naveed, Muhammad Munir, Abdul Ghafoor, Muhammad Naeem Sattar, Hassan Ali-Dinar, Hisham A. Mohamed, Muhammad Asaad Bashir, Muhammad Asif, Adnan Mustafa

**Affiliations:** 1 Institute of Soil and Environmental Sciences, University of Agriculture, Faisalabad, Pakistan; 2 College of Urban and Environmental Science, Northwest University, Xi’an, 710127 China; 3 Date Palm Research Center of Excellence, King Faisal University, Al-Ahsa, 31982 Saudi Arabia; 4 Center for Water and Environmental Studies, King Faisal University, Al-Ahsa, 31982 Saudi Arabia; 5 Central Laboratories, King Faisal University, PO Box 420, Al-Ahsa, 31982 Saudi Arabia; 6 Department of Soil Science, Faculty of Agriculture and Environment, The Islamia University of Bahawalpur, Bahawalpur, Pakistan; 7 Institute of Horticulture, University of Agriculture, Faisalabad, Pakistan; 8 Key Laboratory of Vegetation Restoration and Management of Degraded Ecosystems, South China Botanical Garden, Chinese Academy of Sciences Guangzhou, Guangzhou, 510650 China

**Keywords:** Cadmium, Sunflower, Co-composted biochar, Immobilization, Remediation, Sustainable agriculture

## Abstract

**Background:**

Heavy metals especially cadmium (Cd), has become a matter of concern for environmentalists due to extensive industrialization and poor management of industrial waste. As a toxic pollutant, Cd has ability to deteriorate soil quality and hence disturbs the plant growth and yield. Co-composted biochar (COMBI) has been reported as an excellent organic amendment for improving soil quality, crop productivity and amelioration of heavy metals polluted soil. Therefore, an experiment was performed to assess the potential of co-composted biochar to enhance sunflower growth under Cd stressed soil. Different concentrations 0, 30 and 60 mg kg^-1^ Cd and normal, modified and co-composted biochar at the rate of 1% (w/w) were applied to soil.

**Results:**

The application of normal and modified biochar considerably improved the sunflower growth, yield, physiology and biochemistry and decreased the Cd uptake in plant tissues. Among applied amendments, co-composted biochar showed better results, by increasing the crop agronomic parameters ranging from 115 to 132%, as compared to control treatment under Cd stress. The chlorophyll content, water use efficiency (WUE), photosynthetic rate (A), transpiration rate (E), stomatal conductance (gs), sub-stomatal conductance (Ci), relative water content (RWC), and electrolyte leakage (EL) were improved by 122, 117, 126, 133, 128, 131, 123, and 121%, respectively, when co-composted biochar was used compared to the control. Moreover, stress related metabolites and antioxidant enzyme essays showed increase in proline content, soluble sugars, lipid peroxidation, catalase (CAT), ascorbate peroxidase (APX), superoxide dismutase (SOD) and glutathione reductase (GR) by using co-composted biochar by 123, 121, 118, 128, 124, 133 and 126%, respectively, in Cd (60 mg kg^-1^) contaminated soil. In addition to this, a prominent reduction in accumulation of Cd in the root (66%), shoot (77%) and grain (94%) was observed due to its immobilization in soil (121%) under the influence of co-composted biochar application.

**Conclusion:**

The results of this study revealed that application of biochar could improve crop growth and immobilize Cd in soil and co-composted biochar could be adopted as a better strategy to remediate the heavy metal stressed soils. It can be considered as an effective practical approach to transform agricultural waste materials into organic soil amendments to be applied for sustainable agricultural practices in polluted soil.

## Introduction

The potentially toxic elements (PTEs) contamination particularly heavy metals in the soil have been increased during the last few decades mainly due to the industrial revolution and population explosion, which has consequences for environmental sustainability [1, 2]. Among most hazardous pollutants, heavy metals (HMs) are provoking havoc once introduced into the environment because of their long-term persistence in the environment [3, 4]. The absorption of essential nutrients by crop highly affected by heavy metals accumulation in soil, cause reduction in yield or even failure of crop [5]. Cadmium (Cd) is more dangerous than others heavy metals because it negatively affects the soil characteristics as well as threaten all living beings like plants, animals and especially human life [6]. Anthropogenic sources of Cd present in environment owe to combustion emissions, metal industry, traffic, sewage sludge, mining and incidents [7]. Degraded organic matter and Cd built up in soil disturbs its balance of essential nutrients which affects crop germination, stunts plant growth, and ultimately cause reduction in crop production [8]. Cd in soil and plants reduces the overall growth of plant and productivity mainly due to the interruption of cellular procedures occurring in plants like photosynthesis, respiration, transpiration, stomatal conductance, protein synthesis, antioxidant activity, nutrients uptake etc [9, 10].

In Faisalabad district, Pakistan, chief culprit of high Cadmium (Cd) concentration in soil is industrial discharge causing concentration of Cd by greater than 200% in the soil flooded with this wastewater [11, 12]. From soil, Cd is impacting humans and animal life by bio-magnification [13] causing lethal effects even when present at very low concentrations as it’s a known mutagenic and carcinogenic element [14, 15]. The excessive accumulation of Cd in edible parts of plant is a major challenge to prevent humans from the toxic effects of Cd [16]. Various physical, chemical and biological methods to minimize lethal impacts of HMs in plants have been adopted so far [17]; however, organic amendment’s use have gained great attention owing to their capability to decrease bioavailability of HMs. Among all organic amendments being applied, use of biochar is on the rise due to unique properties it possesses [18, 19].

Biochar is a product of pyrolysis in which organic material produced from animals or plants are heated (350–750 °C) under specific supply conditions of oxygen [20, 21]. It is used to improve the certain properties of soil i.e., soil structure, water and nutrient holding capacity, porosity, water infiltration, pH, organic matter as well as inorganic and organic pollutants removal [22, 23]. Heavy metals (HMs) contamination decreases soil fertility while biochar application increases the organic matter (OM) concentration in soil and ultimately increased fertility of soil [24]. Biochar addition to soil immobilizes the HMs and minimizes their availability and translocation to plants [25], thereby improving soil qualities [26]. Use of biochar amendments in soil improved (Cd) immobilization and decrease its bioaccumulation, ultimately enhance rice yield [27]. Application of biochar on the agricultural land also resulted in an increase of microbial activity to accelerate plant growth and yield by managing stresses like diseases, nutrients leaching and deficiency, mitigation of degraded soil [28, 29]. Under conditions like reduced functional groups, the biochar has low capacity to adsorb contaminants. Some earlier studies have reported that modified sorbent performs more efficiently to adsorb HMs than pristine biochar [18, 30].

Currently, modification procedures for effectively removal of contaminants which is most commonly enhanced by its surface modification with polyethylenimine (PEI). The high adsorption capacity for heavy metals (HMs) owing to presence of amine group [31, 32], are studied to improve sorption ability of biochar [33, 34]. Soil properties as well as physiological and bio**-**chemical features of plant adversely affected by salt stress. However, salt accumulation in soil and plant uptake reduced by residual impacts of microbial modified biochar along with nitrogen (N) fertilizer by improving soil properties as well as plant’s physiological and bio**-**chemical attributes [35]. Activation of biochar by use of potassium hydroxide and sodium hydroxide may produce further C-, H-, O- functional groups like hydroxyl group and increases the sorption capacity by complexation mechanism with enlarged biochar surface area [10, 36]. Application of microbial modified biochar improve the physico-chemical soil properties by immobilization as well as transformation of soil pollutants [37]. Therefore, surface modification of biochar is an important practice to enhance HMs adsorption in soil [38]. The functional group properties of biochar surface can be further augmented by combining with enriched compost product [39, 40]. Compost, usually produced under aerobic conditions through microbial oxidation of crop residues, organic wastes, and animal manure is also used to reclaim polluted soil [41, 42]. The synergistic effect of co-composted biochar (COMBI), which is rarely reported [43, 44], has more organic matter, C content and cation exchange capacity as well, compared to the compost or biochar applied individually [45–47]. It was reported that COMBI could decrease loss of nutrients from agricultural ecosystem and optimize recycling of organic resources for mitigation of climate change, leading to enhance agricultural-productivity [48]. COMBI support soil health, reduce lead uptake in Brassica napus and ultimately improving growth, Physiology as well as biochemical characteristics of plant [49].

Previous studies focused on application of biochar for mitigation of cadmium (Cd) contaminated soil, however, information on how co-composted and modified biochar enhances crop production and minimizes Cd uptake is still lacking. We hypothesized that the co-composted biochar (COMBI) would retrieve the badly effects of Cd and enhance the growth of crops by altering its antioxidants and metabolic profiles. The current study objectives were to (i) evaluate the impacts of normal, modified and co-composted biochar on growth and physiological alterations of sunflower under Cd stress and (ii) assess the capacity of applied amendments to retrieve negative effects of Cd and improve antioxidant, water relations and stress related metabolites of sunflower grown as a test crop under varying levels of Cd.

## Materials and methods

### Preparation of normal, modified, and co-composted biochar

The feedstock material rice husk collected from Institute of Soil and Environmental Sciences (ISES), University of Agriculture Faisalabad (UAF), was ground after drying, and subjected to muffle furnace (Nabertherm B 180, Lilienthal, Germany) at 450 °C pyrolysis temperature [50]. Prepared normal biochar further modified by following study [51, 52]. Prior to modification, normal biochar first treated in 3 mol L^−1^ solution of KOH at the rate of 1:10 (w/v) by adding 20 g biochar with 200 ml solution of KOH in flask and stirred at 160 rpm. at room temperature for a period of 1 h. Subsequently, deionized water was used to rinse biochar until pH of elution reached approximately 7.0. Then biochar dried at 353 K for a period of 12 h and then kept in desiccator before further use. Then KOH treated biochar added with 100 ml of 10% (w/v) polyethyleneimine (PEI) **/** methanol solution and stirred at 160 rpm. and 303 K for 24 h. After this, biochar promptly moved to a 200 ml solution of 1% (w/v) glutaraldehyde for cross linking. The solution stirred at 160 rpm. and 303 K for 30 min. Subsequently, modified biochar was rinsed by using deionized water.

The Co-composted biochar (COMBI) was prepared by mixing 1:2 mass ratio (w/w) of normal biochar and compost respectively during composting process at the compost production unit of ISES. The composting of plant litters (Green Waste) and animal manure was done by using aboveground piles. The plant litters (62%), animals manure (31%) and finished compost material (7%) were firstly placed above ground. The plant litters were laid down initially and animal manure placed on it, then finished compost material spread on it. Then sprayed the pile with water and turned it for 6 times. Now, Biochar was added above pile and turned it for 2 times further. The composts were then aerated by passing plastic perforated pipes through pile. Finally, the piles were covered with plastic sheets. The materials were mixed and sprayed with water at regular intervals and composting process was continued for 2.5 months [53, 54]. The basic characteristics of normal, modified as well as co-composted biochar (COMBI) shown in Table 1.

**Table 1:** The physico-chemical characteristics of Biochar, co-composted Biochar and soil used in present study

Characteristics of biochar	Soil	Normal biochar	Modified biochar	Co-composted biochar
Soil Texture	Sandy clay loam	**-**	**-**	**-**
pH	7.74	7.61	7.45	7.26
Electrical conductivity (dS m^−1^)	1.46	3.4	3.1	3.7
Cation exchange capacity (cmol_c_ kg^−1^)	14.2	78.5	68.3	92.7
Organic matter/organic carbon (%)	0.67	52.35	51.48	50.62
Lime content (%)	2.98	-	-	-
Nitrogen (%)	0.047	1.75	1.64	2.06
Phosphorous (g kg^−1^)	0.38	2.14	2.01	2.36
Potassium (g kg^−1^)	1.19	12.4	9.2	15.7
Calcium (g kg^−1^)	1.62	9.6	8.4	11.7
Magnesium (g kg^−1^)	-	5.1	4.7	6.8
Sulfur (g kg^−1^)	-	2.7	2.5	3.1
Zinc (mg kg^−1^)	-	84.21	65.20	122
Iron (mg kg^−1^)	-	88.34	68.32	162
Manganese (mg kg^−1^)	-	81.22	59.89	145
Cadmium (mg kg^−1^)	ND	-	-	-

*ND* Not detected

### Pot experiment and treatment plan

A pot experiment was carried out in a wire-house at the Institute of Soil and Environmental Sciences (ISES), University of Agriculture Faisalabad (UAF), located at 31**°**26′17″N 73**°**04′09″E. The heavy metals uncontaminated soil (0–20 cm) collected from long term research farm area of ISES. The air-dried, ground and sieved (2 mm mesh size) soil of about 8 kg filled in plastic pots of 10-liter capacity each. The pre-analysis of sieved soil sub-sample was carried out to characterize basic physico-chemical properties (Table 1) through standard methods [55]. Cadmium (Cd) at a rate of 0, 30 and 60 mg kg^−1^ by using salt of cadmium chloride (CdCl_2_) before sowing of sunflower seeds was added to soil. Prepared normal, modified, and co-composted biochar (COMBI) were supplemented (1% w/w). Sunflower variety (Hysun-33) was sown, after thinning, 2 plants pot^−1^ were retained in each pot. Data concerning growth, physiology and yield of plants were recorded at various stages during crop growth.

### Measurement of growth and yield attributes

At harvesting stage, meter rod(cm) was used to measure the shoot and root length of plants after 90 days of sowing. A digital electric balance was used to measure fresh and dry weight of shoot and root. The stem diameter of each plant was measured by using vernier caliper. The head diameter of each plant was taken by measuring the head from one point to the opposite end using a measuring rod. The number of achenes were calculated by counting the achene head^−1^ of each plant and weight of 1000 grains was measured by an electrical balance.

### Physiological parameters

Plant physiological parameters were taken with fully expanded top 2nd leaf at 45th day of plant growth. Portable infrared gas analyzer (IRGA), (Model LCA-4, Germany) at photosynthetic photon flux density of 1200–1400 µmolm^−2^s^−1^ was used for this purpose [56]. Chlorophyll content, i.e., SPAD-502 m (Konica-Minolta, Japan) was used to measure the SPAD (Soil Plant Analysis Development) value [57].The relative water contents (RWC) of sunflower leaves were measured by previous method [58]. For this purpose, fresh, dry, and turgid weight of plant leaves (1 cm^2^) were determined and RWC was calculated by using the formula given below:

Relative water content (%) = $\frac{\mathrm{Fresh}\;\mathrm{wt}.-\mathrm{Dry}\;\mathrm{wt}.}{\mathrm{turgid}\;\mathrm{wt}.-\mathrm{dry}\;\mathrm{wt}.}$ × 100

For determination of electrolyte leakage (EL), cork borer with sharp edge was used to cut leaves into identical discs (1 cm^2^) kept in test tubes (10 ml.) containing distilled water and electrical conductivity (EC1) was recorded. The EC2 was noted by placing these tubes on a mechanical shaker for 2 hours. Afterwards, the tubes were subjected to autoclaving at 120 °C and EC3 was observed upon cooling [59]. Following formula was used to determine EL.

EL (%) = $$\frac{\mathrm{EC}2-\mathrm{EC}1}{\mathrm{EC}3}$$ × 100

Following equation was used to calculate water use efficiency (WUE) [60].

WUE = $$\:\frac{\text{P}\text{h}\text{o}\text{t}\text{o}\text{s}\text{y}\text{n}\text{t}\text{h}\text{e}\text{t}\text{i}\text{c}\:\text{r}\text{a}\text{t}\text{e}\:(\text{A}.)}{\:\text{T}\text{r}\text{a}\text{n}\text{s}\text{p}\text{i}\text{r}\text{a}\text{t}\text{i}\text{o}\text{n}\:\text{r}\text{a}\text{t}\text{e}\:(\text{E}.)\:}$$

### Antioxidant activities

The previously given reduction method by [61] was used to measure superoxide dismutase (SOD) activity and the peroxidase (POD) activity was measured by using the protocol given by [62]. The catalase (CAT) enzyme was estimated by calculating the reduction in H_2_O_2_ absorption at 240 nm [63]. Ascorbate peroxidase (APX) activity was observed by method given below by Ahmad et al. [30] and glutathione reductase (GR) was determined by following [64] method.

### AB-DTPA extractable cd analysis

Available cadmium (Cd) was determined by AB-DTPA method. For this purpose, AB-DTPA solution was prepared by dissolving diethylenetriamine penta acetic acid (DTPA) (1.97 gm) and ammonium bicarbonate (NH_4_)_2_HCO_3_ (79.06 g) and then adjusting the pH to 7.6. After taking 10 g soil in Erlenmeyer flasks, 20 mL of formerly prepared solution was added in it, centrifuged, and transparent extract was filtered, that afterwards was run on atomic absorption spectrophotometer [65].

Metal Cd (mg kg^−1^) = $\:\frac{\text{m}\text{e}\text{t}\text{a}\text{l}\text{s}\:\text{i}\text{n}\:\text{e}\text{x}\text{t}\text{r}\text{a}\text{c}\text{t}\:-\text{m}\text{e}\text{t}\text{a}\text{l}\text{s}\:\text{i}\text{n}\:\text{b}\text{l}\text{a}\text{n}\text{k}}{\text{w}\text{t}.\:\text{o}\text{f}\:\text{s}\text{o}\text{i}\text{l}\:\left(\text{g}\right)}$ × Total volume of extract (ml)

The soil immobilized Cd was calculated by using difference method of soil spiked with Cd (30 and 60 mg kg^−1^) and AB-DTPA extractable Cd.

For Cd analysis in soil and plant tissues, soil/ground plant samples (1 g) were taken in Erlenmeyer flasks and di-acid solution (HNO_3_-HClO_4_) (10 mL) was added in conical flasks that were retained for 24 h and placed on hot plate until the solution turned cleared. After cooling, the samples were filtered and readings were noted using an atomic absorption spectrophotometer [31].

### Remediation indices of cd by sunflower plants

Several indices of remediation efficiency of sunflower plants against Cd were measured such as;

The enrichment factor of Cd metal was calculated as following [66];

$$\:\text{E}\text{n}\text{r}\text{i}\text{c}\text{h}\text{m}\text{e}\text{n}\text{t}\:\text{f}\text{a}\text{c}\text{t}\text{o}\text{r}\:\left(\text{E}\text{F}\right)=\frac{\text{C}\text{d}\:\text{i}\text{n}\:\text{t}\text{h}\text{e}\:\text{g}\text{r}\text{a}\text{i}\text{n}\:}{\text{C}\text{d}\:\text{i}\text{n}\:\text{s}\text{o}\text{i}\text{l}\:}$$

The EF > 1 shows the ability of sunflower plants to store cadmium metal while EF < 1 shows Cd absorption in sunflower plant.

The translocation factor of the Cd metal by sunflower plants was calculated by [67];

$$\:\text{T}\text{r}\text{a}\text{n}\text{s}\text{l}\text{o}\text{c}\text{a}\text{t}\text{i}\text{o}\text{n}\:\left(\text{T}\text{F}\right)=\frac{\text{C}\text{d}\:\text{i}\text{n}\:\text{t}\text{h}\text{e}\:\text{s}\text{h}\text{o}\text{o}\text{t}\:}{\text{C}\text{d}\:\text{i}\text{n}\:\text{r}\text{o}\text{o}\text{t}}$$

Bio accumulation factor (BAF) of sunflower plants was analyzed by [68];

$$\:\text{B}\text{i}\text{o}\:\text{a}\text{c}\text{c}\text{u}\text{m}\text{u}\text{l}\text{a}\text{t}\text{i}\text{o}\text{n}\:\text{f}\text{a}\text{c}\text{t}\text{o}\text{r}\:\left(\text{B}\text{A}\text{F}\right)=\frac{\:\text{C}\text{d}\:\text{i}\text{n}\:\text{t}\text{h}\text{e}\:\text{r}\text{o}\text{o}\text{t}\:}{\text{C}\text{d}\:\text{i}\text{n}\:\text{s}\text{o}\text{i}\text{l}\:}$$

Bio accumulation coefficient (BAC) was calculated as described by [69];

$$\:\text{B}\text{i}\text{o}\:\text{a}\text{c}\text{c}\text{u}\text{m}\text{u}\text{l}\text{a}\text{t}\text{i}\text{o}\text{n}\:\text{c}\text{o}\text{e}\text{f}\text{f}\text{i}\text{c}\text{i}\text{e}\text{n}\text{t}\:\left(\text{B}\text{A}\text{C}\right)=\frac{\:\text{C}\text{d}\:\text{i}\text{n}\:\text{t}\text{h}\text{e}\:\text{s}\text{h}\text{o}\text{o}\text{t}\:}{\text{C}\text{d}\:\text{i}\text{n}\:\text{s}\text{o}\text{i}\text{l}}$$

### Cd health risk assessment parameters

Several indices for health risk assessment were calculated as mentioned below;

The average daily intake (ADI) index of cadmium (Cd) metal determined by using method given below;

$$\:\text{A}\text{v}\text{e}\text{r}\text{a}\text{g}\text{e}\:\text{D}\text{a}\text{i}\text{l}\text{y}\:\text{i}\text{n}\text{t}\text{a}\text{k}\text{e}\:\left(\text{A}\text{D}\text{I}\right)=\frac{\:\text{M}\times\:\text{I}}{\text{W}}$$

The Cd concentration in plant, daily intake of sunflower and average body weight (BW) denoted by M, I and W respectively in above equation. The adults have an average BW of 60 kg with average daily intakes of sunflower were considered 0.345 kg person^−1^ day^−1^ [70].

The Cd non-cancer risk (NCR) Cd was due to consumption of contaminated sunflower, calculated as [71];

$$\:\text{N}\text{o}\text{n}\:\text{C}\text{a}\text{n}\text{c}\text{e}\text{r}\:\text{R}\text{i}\text{s}\text{k}\:\left(\text{H}\text{Q}\right)=\frac{\:\text{A}\text{D}\text{I}\:}{\text{R}\text{F}\text{D}}$$

Oral reference doses (RFD) for Cd, is 0.5 [72].

Following formula to calculate integrated lifetime cancer risk (ILTCR), through ingestion of Cd contaminated grain [73];

$$\:CR\:=ADI\times\:CSF\:$$

Where “CSF” represents the oral cancer slope factor for metal. For this study, cadmium, has CSF of 0.38 [74].

### Statistical analysis

The Analysis of variance (ANOVA) test was used to analyzed the data obtained and means were compared with Tukey’s honestly significant difference (HSD) test for significant differences among different applied treatments at 5% probability level using Statistix 8.1 (Analytical software, 2005) [75]. The correlation plot was plotted in Origin Pro software and PCA plot was constructed with R studio software.

## Results

### Impact on plant growth and yield

**Fig. 1:**
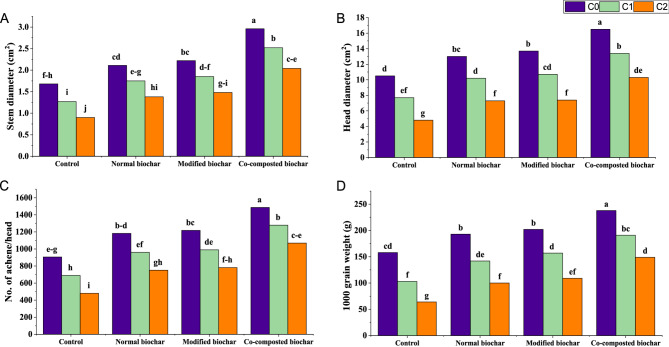
Effect of normal, modified, and co-composted biochar on yield attributes i.e., (**A**) stem diameter, (**B**) head diameter, (**C**) No. of achene/head, (**D**) 1000 grain weight of sunflower in Cd spiked soil. Here, C0, C1, and C2 indicate Cd 0, 30 and 60 mg kg^−1^, respectively

Plant growth and yield parameters were remarkably affected by Cadmium (Cd). Cd concentration at 60 mg kg^−1^ negatively affected growth of plant (Table 2) and yield (Fig. 1) attributes. However, application of both normal and modified biochar significantly improved the plant growth attributes at elevated Cd toxicity (30 and 60 mg kg^−1^), compared to control treatment. Furthermore, co-composted biochar (COMBI) addition significantly improved plant height (128%), root length (120%), fresh weight of shoot (127%) and root (121%), dry weight (DW) of shoot (126%) and root (118%), stem diameter (126%), head diameter (115%), no. of achene head^−1^ (123%), and 1000 grain weight (132%) at 60 mg kg^−1^ Cd over treatment set as control.

**Table 2:** Effect of normal, modified, co-composted Biochar on growth attributes of sunflower under cadmium contaminated soil

Cadmium	Amendment	Plant height (cm)								Fresh weight (g)								Dry weight (g)							
(mg kg^−1^)		Shoot				Root				Shoot				Root				Shoot				Root			
0	Control	75 ± 3.66			de	47.6 ± 1.58			d	95 ± 4.23			de	58.7 ± 4.33			d-f	12.4 ± 0.63			d-f	9.4 ± 0.35			de
	Normal biochar	91 ± 1.92			bc	57.9 ± 1.37			bc	120 ± 4.81			bc	76.2 ± 3.03			bc	15.9 ± 0.78			bc	12.1 ± 0.41			bc
	Modified biochar	99 ± 3.65			b	60.3 ± 1.30			bc	130 ± 4.23			B	83.1 ± 2.87			ab	17.0 ± 0.66			b	13.4 ± 0.47			b
	Co-composted biochar	115 ± 3.87			a	69.3 ± 1.56			a	151 ± 6.46			A	99.9 ± 4.54			a	21.3 ± 0.52			a	16.0 ± 0.59			a
30	Control	54 ± 2.41			f	37.7 ± 2.26			e	76 ± 3.99			ef	42.9 ± 3.12			f	9.6 ± 0.43			f	7.2 ± 0.47			e-g
	Normal biochar	75 ± 3.76			de	49.5 ± 2.01			d	98 ± 2.44			D	61.0 ± 3.19			c-e	12.9 ± 0.48			c-e	9.2 ± 0.48			d
	Modified biochar	83 ± 1.29			cd	53.0 ± 0.93			cd	103 ± 2.97			cd	65.8 ± 2.58			cd	13.8 ± 0.55			cd	10.7 ± 0.54			cd
	Co-composted biochar	98 ± 1.93			b	62.2 ± 1.83			ab	124 ± 2.44			B	83.9 ± 3.27			ab	17.1 ± 0.57			b	13.2 ± 0.58			b
60	Control	34 ± 2.26			g	21.8 ± 1.82			f	41 ± 4.23			G	29.8 ± 2.42			g	6.0 ± 0.65			g	4.7 ± 0.45			h
	Normal biochar	51 ± 1.25			f	33.1 ± 1.05			e	62 ± 3.99			F	43.5 ± 1.98			f	9.5 ± 0.62			f	6.9 ± 0.40			fg
	Modified biochar	62 ± 3.23			ef	38.6 ± 1.13			e	72 ± 4.23			F	47.8 ± 3.44			ef	10.1 ± 0.76			e-f	7.6 ± 0.51			ef
	Co-composted biochar	77 ± 4.02			cd	48.0 ± 2.06			d	93 ± 2.44			de	65.9 ± 4.10			cd	13.6 ± 0.69			cd	10.1 ± 0.59			cd

The values are mean ± S.E. (*n* = 3). Means sharing similar letter(s) in a column for each parameter do not differ significantly at *P* = 0.05

### Impact on crop physiology

Cadmium (Cd) at 60 mg kg^−1^ extremely decreased chlorophyll content (16.4 mg cm^−2^), A (8.02 µmol m^−2^ s^−1^), E (1.79 mmol m^−2^ s^−1^), gs (60 mmol m-2 s-1), Ci (107 µmol mol^−1^) as shown in Table 3. However, the application of both Normal and modified biochar expressively improved the plant physiological parameters under increased Cd toxicity levels of 30 and 60 mg kg^−1^ compared to the control treatment. Nevertheless, co-composted biochar (COMBI) application considerably better enhanced the chlorophyll content (122%), A (126%), E (133%), gs (128%), and Ci (131%) at 60 mg kg^−1^ Cd.

**Table 3:** Effect of normal, modified, co-composted Biochar on physiological attributes of sunflower under cadmium contaminated soil

Cadmium	Amendment	Photosynthesis				Transpiration				Stomatal				Sub-stomatal				Relative water				Electrolyte				Chlorophyll Contents		Water Use	
		rate (A)				rate (E)				conductance (gs)				conductance (Ci)				content (RWC)				leakage (EL)						Efficiency (WUE)	
(mg kg^−1^)		(µmol m^−2^ s^−1^)				(mmol m^−2^ s^−1^)				(mmol m^−2^ s^−1^)				(µmol mol^−1^)				(%)				(%)				(mg cm^−2^)		(%)	
0	Control	15.3 ± 0.87			De	3.91 ± 0.19			de	122 ± 3.33			de	210 ± 8.83			Ef	50.1 ± 2.32			d	5.83 ± 0.23			Cd	35 ± 1.9	d	2.11 ± 0.05	ef
	Normal biochar	19.9 ± 0.58			Bc	5.01 ± 0.2			bc	157 ± 4.54			bc	284 ± 9.91			b-d	61.5 ± 2.34			bc	3.36 ± 0.17			Fg	42 ± 1.27	bc	2.75 ± 0.08	bc
	Modified biochar	21.2 ± 0.98			bc	5.43 ± 0.24			b	174 ± 5.90			b	290 ± 11			Bc	65.1 ± 2.50			b	3.25 ± 0.15			Fg	44 ± 1.21	b	2.88 ± 0.06	b
	Co-composted biochar	26.1 ± 0.7			A	6.51 ± 0.25			a	202 ± 7.34			a	373 ± 13.1			A	76.6 ± 1.15			a	1.56 ± 0.12			H	53 ± 1.21	a	3.46 ± 0.07	a
30	Control	11.7 ± 0.53			F	2.84 ± 0.1			F	91 ± 3.40			f	159 ± 7.51			G	36.5 ± 2.09			e	7.54 ± 0.22			B	28 ± 1.54	ef	1.6 ± 0.07	g
	Normal biochar	16 ± 0.54			D	3.9 ± 0.12			de	123 ± 4.34			de	227 ± 8.66			Ef	49.5 ± 2.38			d	4.90 ± 0.20			De	34 ± 1.33	de	2.24 ± 0.07	de
	Modified biochar	16.5 ± 0.54			D	4.24 ± 0.2			cd	140 ± 5.34			cd	235 ± 9.24			De	51.4 ± 2.41			cd	4.39 ± 0.17			E	37 ± 0.86	cd	2.45 ± 0.06	cd
	Co-composted biochar	21.9 ± 0.58			B	5.35 ± 0.2			b	170 ± 5.65			b	310 ± 9.82			B	62.6 ± 2.26			b	2.78 ± 0.12			G	45 ± 1.32	b	3.01 ± 0.04	b
60	Control	8.02 ± 0.35			G	1.79 ± 0.16			g	60 ± 4.18			g	108 ± 6.14			H	22.3 ± 2.31			f	9.14 ± 0.25			A	16 ± 1.02	g	1.15 ± 0.06	h
	Normal biochar	11.8 ± 0.58			F	2.81 ± 0.2			f	91 ± 5.46			f	180 ±9.05			Fg	35.3 ± 1.25			e	6.40 ± 0.23			C	24 ± 1.37	f	1.73 ± 0.04	g
	Modified biochar	12.7 ± 0.64			ef	3.12 ± 0.25			ef	107 ± 6.77			ef	185 ± 10.1			Fg	37.2 ± 1.48			e	5.99 ± 0.16			C	26 ± 1.16	f	1.87 ± 0.08	fg
	Co-composted biochar	18.1 ± 0.45			cd	4.17 ± 0.22			cd	138 ± 7.13			cd	250 ± 12.1			c-e	49.7 ± 2.08			d	4.14 ± 0.12			Ef	36 ± 1.62	cd	2.5 ± 0.06	cd

The values are mean ± S.E. (*n* = 3). Means sharing similar letter(s) in a column for each parameter do not differ significantly at *P* = 0.05

Water relation parameters (Table 3) were also affected by toxicity of Cd at elevated level (30 and 60 mg kg^−1^). In control treatment, Cd at 60 mg kg^−1^ level, decreased the WUE (1.15%), RWC (22.3%), and EL (9.14%). Application of normal and modified biochar efficiently enhanced the plant physiological parameters under Cd toxicity (30 and 60 mg kg^−1^) over control treatment. Here, application of COMBI further enhanced WUE (117%), RWC (123%), and decreased the EL (121%) under Cd stressed soil.

### Antioxidant activities

**Fig. 2:**
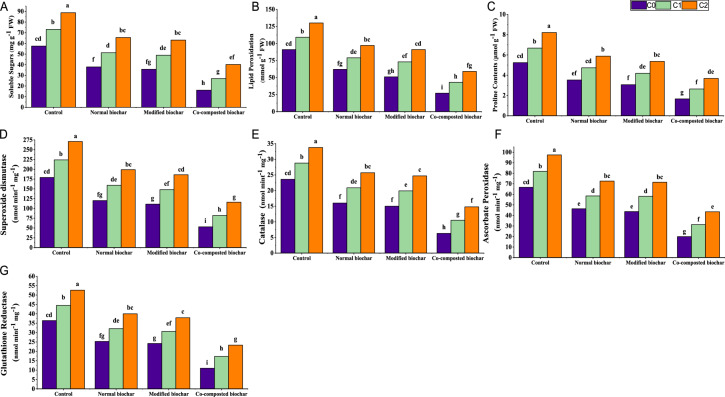
Effect of normal, modified, and co-composted biochar on sunflower’s antioxidant activities i.e., (**A**) soluble sugar, (**B**) lipid peroxidation, (**C**) proline contents, (**D**) superoxide dismutase, (**E**) catalase (**F**) ascorbate peroxidase, (**G**) Glutathione reductase, in Cd spiked soil. Here, C0, C1, and C2 indicate Cd 0, 30 and 60 mg kg^−1^, respectively

Under Cadmium (Cd) contaminated soil, the stress-related metabolites including, proline content, soluble sugar, lipid peroxidation and activity of antioxidant enzymes (CAT, APX, SOD and GR) significantly increased as shown in Fig. 2. However, stress-related metabolites and antioxidant activities were significantly declined by addition of normal and modified biochar. While, application of co-composted biochar (COMBI) showed substantial better results and decreased the proline content (123%), soluble sugars (121%), lipid peroxidation (118%), CAT (128%), APX (124%), SOD (133%), and GR (126%) over control treatment in Cd contaminated soils.

### Cd content in soil and plant tissues

**Fig. 3:**
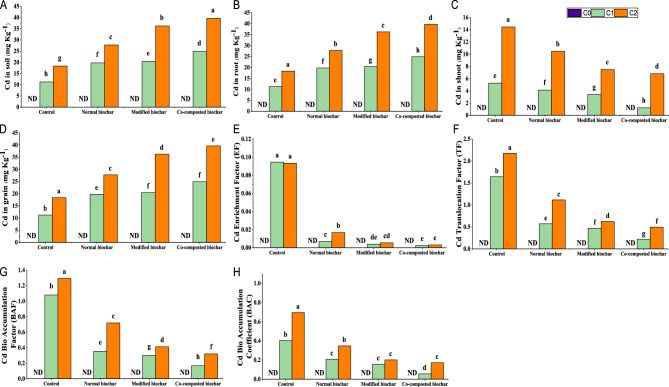
Effect of normal, modified, and co-composted biochar on Cd concentration in sunflower i.e., (**A**) Cd in soil, (**B**) Cd in root, (**C**) Cd in shoot, (**D**) Cd in grain, (**E**) Cd enrichment factor, (**F**) Cd translocation factor, (**G**) Cd bio accumulation factor, (**H**) Cd bio accumulation coefficient, in Cd spiked soil. Here, C0, C1, and C2 indicate Cd 0, 30 and 60 mg kg^−1^, respectively

Concentration of cadmium (Cd), (Fig. 3) in the soil was observed at 11.2 and 18.3 µg g^−1^ and most of the Cd was accumulated in root (12.1 and 23.7 µg g^−1^), shoot (5.2 and 14.5 µg g^−1^) and grain (1.06 and 1.7 µg g^−1^) at 30, and 60 mg kg^−1^ Cd, respectively in control. However, application of both normal and modified biochar significantly immobilized Cd in soil and decreased its accumulation in aerial parts (root and shoot) of the plant. Whereas co-composted biochar (COMBI) significantly increased the Cd presence in soil (121%) and decreased Cd concentrations in root (66%), shoot (77%) and grains (94%) over control under 30 mg kg^−1^ Cd toxicity.

### Cd phytoremediation

The data obtained from the cadmium (Cd) phytoremediation by analyzing parameters of bio accumulation factor, translocation factor, and bio accumulation coefficient for Cd (Fig. 3E, F, G, H). The least values of 0.002 for EF, 0.29 for TF, 0.17 for BAF, and 0.055 for BAC were observed in presence of co-composted biochar in sunflower supplemented with 30 mg kg^−1^ cadmium.

### Health risk assessment

Health risk assessment parameters of sunflower plants showed minimum values 0.003 for ADI, 0.30 for NCR, and 0.00011 for CR in plants treated with co-composted biochar cultivated with 30 mg kg^−1^ cadmium contaminated soil (Table 4).

**Table 4:** Normal, modified, co-composted biochar’s effects on health risk assessment of sunflower under cadmium contaminated soil

Cadmium	Amendment	Health Risk Assessment					
(mg kg^−1^)		ADI		NCR	CR		
0	Control	0	0		0		
	Normal biochar	0	0		0		
	Modified biochar	0	0		0		
	Co-composted biochar	0	0		0		
30	Control	0.0052 b	5.24 b		0.00199 b		
	Normal biochar	0.0007 e	0.68 e		0.00026 e		
	Modified biochar	0.0004 f	0.39 f		0.00015 f		
	Co-composted biochar	0.0003 f	0.30 f		0.00011 f		
60	Control	0.0084 a	8.42 a		0.00320 a		
	Normal biochar	0.0023 c	2.2 c		0.00087 c		
	Modified biochar	0.0010 d	0.96 d		0.00037 d		
	Co-composted biochar	0.0006 e	0.61 e		0.00023 e		

The values are mean ± S.E. (*n* = 3). Means sharing similar letter(s) in a column for each parameter do not differ significantly at *P* = 0.05

### Pearson correlation and principal component analysis (PCA.)

**Fig. 4:**
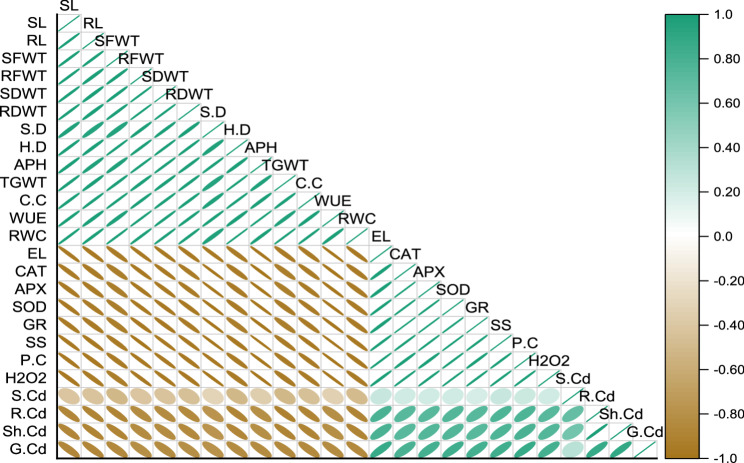
Pearson correlation between parameters of sunflower plants under application of normal, modified, and co-composted biochar grown in Cd spiked soil. Here parameters are indicated as SL = shoot length, RL = root length, SFWT = shoot fresh weight, RFWT = root fresh weight, SDWT = shoot dry weight, RDWT = root dry weight, SD = stem diameter, HD = head diameter, APH = number of achene per head, TGWT = thousand grain weight, RWC = relative water contents, EL = electrolyte leakage, CC = chlorophyll contents, WUE = water use efficiency, SS = soluble sugars, H2O2 = lipid peroxidation, P.C = proline contents, SOD = superoxide dismutase, CAT = catalase, APX = ascorbate peroxidase, GR = glutathione reductase, S.Cd = Cd in soil, RCd = Cd in root, Sh.Cd = Cd in shoot, GCd = Cd in gain

Significant positive as well as negative correlations (Fig. 4) were analyzed among the plant growth (length, fresh and dry weight of root and shoot), yield (head and stem diameter, no. of achene per head and 1000 grain weight), physiology (relative water contents, chlorophyll contents, water use efficiency), biochemical (soluble sugars, lipid peroxidation, proline, sodium dismutase, catalase, ascorbate peroxidase and glutathione), and heavy metal contents (soil, root, shoot, grain, EF, TF, BAF, BAC) of treatments under cadmium (Cd) stress and application of amendments. The SL, RL, SFWT, RFWT, SDWT, RDWT, SD, HD, APH, TGWT, RWC, CC, WUE, showed positive correlation with each other indicating that these parameters showed improved activity in presence of amendments under Cd stress. However, growth, physiology and yield parameters showed negative correlation with biochemical and heavy metals related parameters like EL, SS, H2O2, PC, SOD, CAT, APX, GR, S.Cd, R.Cd, Sh.Cd, and G.Cd.

**Fig. 5:**
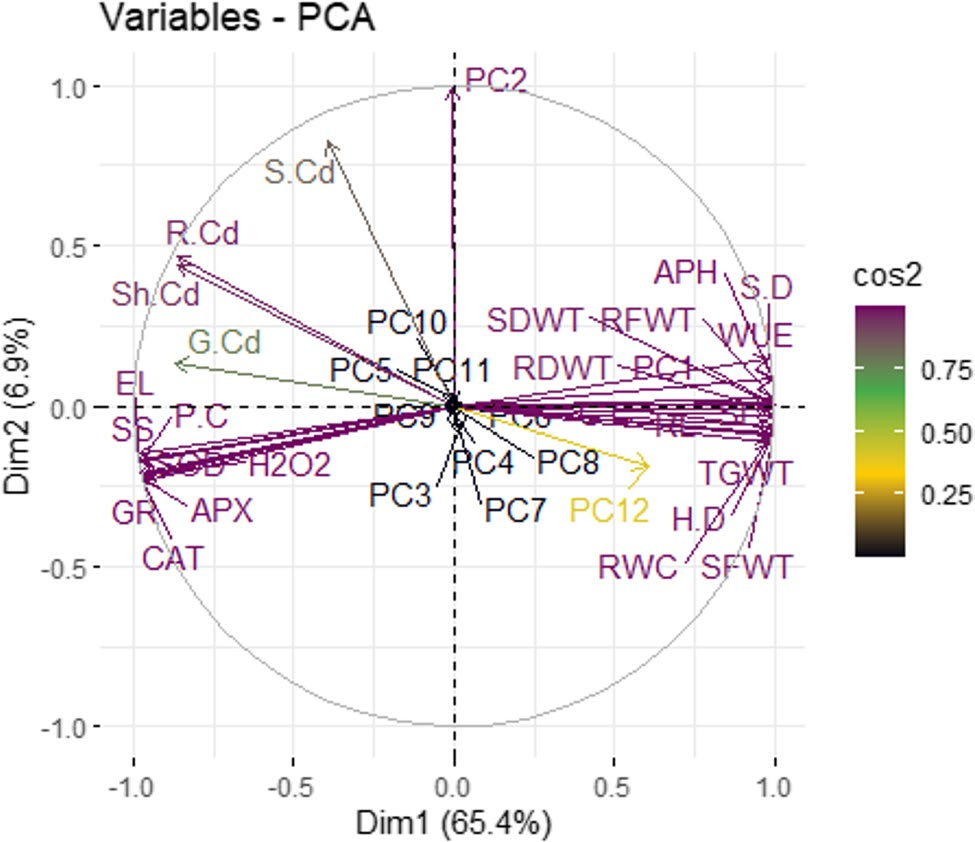
Principal component analysis (PCA) between parameters of sunflower plants under application of normal, modified, and co-composted biochar grown in Cd spiked soil. Here parameters are indicated as SL = shoot length, RL = root length, SFWT = shoot fresh weight, RFWT = root fresh weight, SDWT = shoot dry weight, RDWT = root dry weight, SD = stem diameter, HD = head diameter, APH = number of achene per head, TGWT = thousand grain weight, RWC = relative water contents, EL = electrolyte leakage, CC = chlorophyll contents, WUE = water use efficiency, SS = soluble sugars, H2O2 = lipid peroxidation, P.C = proline contents, SOD = superoxide dismutase, CAT = catalase, APX = ascorbate peroxidase, GR = glutathione reductase, S.Cd = Cd in soil, RCd = Cd in root, Sh.Cd = Cd in shoot, GCd = Cd in grain

The principal component analysis (PCA) revealed (Fig. 5) that shoot length (SL), root length (RL), fresh weight of shoot (SFWT) and root (RFWT), dry weight of shoot (SDWT) and root (RDWT), stem diameter (SD), head diameter (HD), achene per head (APH), thousand grain weight (TGWT), relative water contents (RWC), chlorophyll contents (CC) and water use efficiency (WUE), indicated strong correlated among parameters due to application of amendments under Cd toxicity. Whereas, growth, physiology and yield parameters showed negative correlation with biochemical and heavy metals related parameters like electrolyte leakage (EL), soluble sugars (SS), lipid peroxidation (H2O2), proline (PC), sodium dismutase (SOD), catalase (CAT), (ascorbate peroxidase (APX), glutathione (GR), Cd in soil (S.Cd), root Cd (RCd), shoot Cd (SCd) and Cd in grain (GCd).

## Discussion

Heavy metals have gained a significant attention from researchers due to their persistent and hazardous nature, having capacity to localized in plant parts and therefore disturbing agroecosystem and risking health of living organisms [30]. The crop production limited by number of factors, drought, salinity, chilling stress and heavy metals toxicity as described by researchers [9, 76]. Among heavy metals, cadmium (Cd) is more harmful for human, plant, and animal health due to its mobility in soil [6, 77].

The growth of sunflower plants was limited due to contamination of soil with cadmium at both 30 and 60 mg kg^−1^ levels. Declined in the length of root and shoot as well as fresh and dry weight, were observed with application of 30 and 60 mg kg^−1^ cadmium. The presence of Cd in soil interrupts the nutrient uptake by plants, therefore, causing nutrients deficiency. Similar to this study previously, with increased application of metal contaminated textile wastewater, plant growth in tomato plants was declined [78].

However, cadmium (Cd) stress was remediated effectively by applying normal, modified, and co-composted biochar (COMBI) to improve the growth of sunflower in Cd spiked soil. The current study showed significant increase in lengths, fresh and dry weight of root and shoot of sunflower plants with addition of normal, modified, and co-composted biochar, compared to control (without amendments) grown in soil contaminated with Cd. Biochar is a source of various micro and macro nutrients and hence beneficial in soils with nutrients deficiency. Hence, improves the fertility of soil as reported by [79]. Large surface area of biochar was also beneficial in holding Cd metal in soil. Therefore, the increase growth was attributed to the provision of nutrients, as well as immobilization and retention of soluble Cd by biochar, coalesce with the findings of [80]. However, overall significant growth was noticed when COMBI was supplied to sunflower in contrast to normal and modified biochar under normal and Cd stressed soils [81].

The presence of cadmium (Cd) resulted in reduced diameter of stem and head, number of achenes per head and thousand grain weight, therefore, causing reduction in yield of sunflower plants. Declined yield of sunflower plants exposed to Cd, might be linked with harmful effects of Cd [82]. High concentration of Cd in soil probably has disrupted soil characteristics and have replaced the essential nutrient required for proper growth of plant and eventually resulted in limiting yield [83]. Whereas application of amendments most importantly co-composted biochar (COMBI) provided the desired nutrients to the normal functioning of plants while remediating cadmium metal [9]. Overall comparison revealed that yield parameters were increased maximum by using COMBI than modified and normal biochar. Therefore, it might be considered as an effective methodology to be applied for sustainable agricultural practices in Cd stress [76, 84].

Cadmium decreased the physiological parameters in sunflower plant, which ultimately declined plant growth [16, 85, 86]. Furthermore, declined CO_2_ assimilation is associated with decreases production of carboxylic enzymes [87] and Rubisco activity [88]. Therefore, data of the current study correlates with previous studies, indicating that Cd causes a reduction in photosynthetic pigments, the number of stomata, and other physiological traits, thereby exerting a negative effect [19, 20]. The study results revealed a decline in sunflower efficiency regarding physiological processes, when exposed to Cd.

When plant was exposed to cadmium (Cd) stress, parameters like WUE and RWC decreased. Reduction in chlorophyll pigment decreased the photosynthetic and transpiration rate in plants which may lead to decrease in WUE. According to [89], Cd retards the physiological mechanisms like photosynthesis and transpiration in plants. Whereas, by the application of biochar, increase in WUE and RWC parameters is associated with the improved growth as well as crop yield. Moreover, EL was also increased due to membrane damage Cd phytotoxicity [90]. The supplementation of normal and surface modified biochar caused significant decrease in Cd levels in soil, while use of co-composted biochar (COMBI) led to an even more substantial reduction. This effect might enhance Cd immobilization in soil and reduced uptake by plants. Conversely, prior research has reported a significant increase in these physiological attributes through the utilization of biochar [91, 92]. The physiological traits were more significantly increased by addition of modified biochar, but application of COMBI showed much better and significant results over all the applied treatments including control. The biochar and compost application synergistically immobilized Cd in the soil and reduced harmful effects because of their ability to make metals complex, and decreased Cd bioavailability resulting in improved physiological attributes of crops [93].

Sunflower plants exposed to the cadmium (Cd), showed significant reduction in antioxidant activities. Increased metal levels in plants leads to disruption in DNA, producing reactive oxygen species, and hence damaging the biomolecules [11]. Moreover, translocation of Cd metal in plant tissues causes altered function of essential nutrients and therefore, generating reactive oxygen species (ROS) [90]. However, application of normal, modified, and co-composted biochar significantly improved the production of antioxidants in sunflower in contrast to control under the toxicity of Cd that showed their progressive role in reducing toxicity of Cd [11].

Increased immobilization of cadmium metal was found in soil polluted with different cadmium (Cd) concentrations (30, 60 mg Kg^−1^), in presence of co-composted biochar (COMBI). Whereas increased Cd level was reported in roots as compared to other plant parts. Plants highly differ in their biogeochemical behavior to buildup Cd in underground and above ground parts as well as in transportation of metal to root through soil that largely depends upon accessible soil portion of metal and in concern of roots to shoots vary with species and genotype of the tested plant [94–96]. In this study, Cd buildup into various plant parts was observed due to intentional addition of Cd to the soil as an exogenous source. These findings are in accordance with the previously reported literature [97]. Moreover, large quantity of root exudates production which binds Cd by cell wall and extracellular medium led to buildup of their larger concentration in root [98]. The Cd bioaccumulation depends upon its binding capacity, intercellular complex as well as its transportation within various plant parts [99–101]. Furthermore, concentration of the Cd in plant highly differs with its capacity for intercellular buildup of CO_2_ and transpiration. The findings of current study elucidated that exposure to the Cd exerted substantial detrimental effects on the stomatal conductance, transpiration rate, and also sub-stomatal conductance of the investigated crop. This phenomenon likely facilitated the enhanced accumulation of Cd in sunflower plants via soil contamination. Nevertheless, the utilization of co-composted, modified, and normal biochar proved efficacious in mitigating these adverse effects by instigating a mechanism of Cd immobilization within the soil, thereby reducing its concentration in the soil-plant continuum. Notably, stomatal conductance and transpiration rate are integral factors governing the dynamic transport and uptake of both essential nutrients and contaminants, including Cd, within the intricate soil-plant-atmosphere interface [60]. However, reduced Cd was transported and stored in other plant parts shoot and grains respectively. The declined cadmium uptake was also indicated by least values of EF, TF, BAF, and BAC along with reduced ADI, NCR, and CR for sunflower grown in Cd contaminated soils in presence of co-composted biochar (COMBI). The reduction of Cd uptake and translocation to upper parts of plant is also a part of adaptive strategy in plants growing in soil contaminated with metals [77].

Use of amendments especially co-composted biochar (COMBI) showed increased cadmium (Cd) concentration in soil while declined metal uptake was observed in root, shoot and also grains of sunflower plant sown in soil contaminated with Cd. The biochar has the unique remediation properties like presence of large surface area, macro and micro pores, binding sites and presence of various functional groups that are key factors in holding Cd in soil and hence reducing uptake and translocation of the Cd within plant tissue. The increased immobilization of metals like Cd is reported previously by researchers [102, 103]. This study revealed, addition of normal, modified, and COMBI significantly stabilized Cd in soil and reduced their bioavailability to crop which may lead to significant improvement in the growth as well as physiological factors. However, efficiency of these factors more significantly improved by use of COMBI in contrast to modified and normal biochar. Because, in addition to biochar, compost also contains different functional group on its surface and an immediate as well as rich source of nutrients which increases the effectiveness of biochar in regard to growth, physiology as well as Cd immobilization in soil. Furthermore, COMBI use resulted in significantly more decline in bioavailable fractions of soil’s Cd and decreased its concentration in plant [104] as compared to normal and modified biochar.

From the above discussion, it reveals that Cadmium (Cd) toxicity cause serious threats to soil-plant system. Whereas co-composted biochar (COMBI) has significant potential for crop growth and amelioration of Cd polluted soil. Cd bioavailability in the soil significantly reduced by the application of (COMBI) and ultimately decreased its accumulation in sunflower plant tissues. The capacity of soil to hold water and nutrients increased by the application of COMBI which improves root development along with plant health which eventually results in higher plant biomass and yield. It restricts the entering of Cd into food chain as well as improve crop safety along with marketability. By Using COMBI as an amendment in the Cd polluted soil, farmers can grow crops safely which are susceptible to stress of heavy metals. It promotes improvement in structure, organic matter contents as well as soil microbial properties of soil, leading to sustainability in agriculture. In this way, farmers operating on heavy metals polluted fields can get good economics outcomes from higher yields. This approach is adaptable and farmers can use COMBI as an effective organic soil amendment for sustainable agricultural practices.

## Conclusion

The study demonstrates that co-composted biochar is a highly effective amendment for mitigating cadmium (Cd) stress in sunflower, significantly improving plant growth, physiological performance, and antioxidant responses. Under Cd-contaminated soil conditions, co-composted biochar outperformed normal and modified biochar by enhancing key agronomic parameters, such as root and shoot growth, yield, and physiological traits like chlorophyll content, water use efficiency, and photosynthetic rate. It also boosted antioxidant enzyme activities (CAT, APX, SOD, GR) and stress-related metabolites, reducing oxidative damage caused by Cd toxicity. Importantly, co-composted biochar immobilized Cd in the soil, reducing its uptake in roots, shoots, and grains by up to 94%, thereby minimizing health risks associated with Cd accumulation. The synergistic effects of biochar and compost in co-composted biochar not only improved soil fertility and nutrient availability but also enhanced Cd immobilization through its unique physicochemical properties, such as large surface area and functional groups. This study highlights the potential of co-composted biochar as a sustainable and eco-friendly strategy for the remediation of heavy metal-contaminated soils, promoting crop productivity, and ensuring food safety.

## Data Availability

Data is provided within the manuscript or supplementary information files.
