# Correction: Revitalizing cadmium-stressed sunflower: co-composted Biochar improves growth, antioxidant responses, and soil remediation efficiency

**DOI:** 10.1186/s12870-025-07098-1

**Published:** 2025-07-29

**Authors:** Muhammad Rauf, Muhammad Naveed, Muhammad Munir, Abdul Ghafoor, Muhammad Naeem Sattar, Hassan Ali-Dinar, Hisham A. Mohamed, Muhammad Asaad Bashir, Muhammad Asif, Adnan Mustafa

**Affiliations:** 1 Institute of Soil and Environmental Sciences, University of Agriculture, Faisalabad, Pakistan; 2 College of Urban and Environmental Science, Northwest University, Xi’an, 710127 China; 3 Date Palm Research Center of Excellence, King Faisal University, Al-Ahsa, 31982 Saudi Arabia; 4 Center for Water and Environmental Studies, King Faisal University, Al-Ahsa, 31982 Saudi Arabia; 5 Central Laboratories, King Faisal University, PO Box 420, Al-Ahsa, 31982 Saudi Arabia; 6 Department of Soil Science, Faculty of Agriculture and Environment, The Islamia University of Bahawalpur, Bahawalpur, Pakistan; 7 Institute of Horticulture, University of Agriculture, Faisalabad, Pakistan; 8 Key Laboratory of Vegetation Restoration and Management of Degraded Ecosystems, South China Botanical Garden, Chinese Academy of Sciences, Guangzhou, 510650 China

**Correction: BMC Plant Biol 25, 875 (2025)**

https://doi.org/10.1186/s12870-025-06906-y

Following publication of the original article [1], the authors would like to update the Authors’ contribution section.

**Current authors’ contribution**

Conceptualization, M.N., A.M.; methodology, M.A.B., Z.N., and M.R.; software, M.R. and A.G.; formal analysis, A.M., M.M., and M.N.; investigation, M.A. B., M.R.; resources, M.N., M.M.,.; data curation, M.R., M.I.K., M.R.; writing—original draft preparation, M.R. and M.N.; writing—review and editing, A. M., M.S.A., A.G.,.; visualization, A.M., supervision, M.N. All authors have read and agreed to the published version of the manuscript.

**Updated authors’ contribution**

Conceptualization, M.N. and A.M.; methodology, M.R. and M.A.B.; software, M.R. and A.G.; formal analysis, A.M., M.M. and M.N.; investigation, M.A.B. and M.R.; resources, M.N., M.M., H.A.D. and H.A.M.; data curation, M.R. and M.A.; writing-original draft preparation, M.R. and M.N.; writing-review and editing, A. M., M.N.S. and A.G.; visualization, A.M., supervision, M.N.

The correction does not compromise the validity of the conclusions and the overall content of the article. The author group has been updated above and the original article [1] has been corrected.
